# Austin District Medical Association

**Published:** 1887-09

**Authors:** 


### AUSTIN DISTRICT MEDICAL SOCIETY.

"AND NOW BY SAINT GEORGE, THE GOOD WORK GOES BRAVELY ON" (or words to that effect;) "Another precinct heard from."

(COMMUNICATED.)

The question of organizing a district medical association has been agitated for some time, and as an outcome some thirty physicians met yesterday at the hall of the Travis County Medical Society and organized. All the adjoining counties were represented, and the meeting was one full of interest, and the prospect of an enthusiastic and useful organization is very promising.

Prominent amongst the visiting physicians were Drs. Black, of Round Rock; Gregg and Richmond, of Manor; Cocke, of San Marcos; Ellison and Davidson, of Manchaca; Hamilton of Hornsby; Cunningham, of Elgin; Hardy, of Paige; F. A. Maxwell, of Fiskville, and others. The physicians of Austin were largely represented. Dr. W. A. Morris was called to the chair, and Dr. Q. C. Smith requested to act as secretary.

A constitution and by-laws were adopted, and then the meeting went into permanent organization by the election of officers.

Dr. T. D. Wooten was elected president, and Dr. W. T. Richmond, of Manor, first vice-president; Dr. L. H. Hardy, of Paige, second vice-president, Dr. T. J. Bennett, secretary. The following gentlemen were elected a board of censors: Drs. Swearingen, Denton, Black, Cunningham and Ellison.

The next meeting will be held in Austin, second Thursday in December (eighth day)—it being determined to meet quarterly. Doctors Bennett, Black, Cocke and Cunningham were appointed to read essays at next meeting. It is understood that in addition to the regular essays there will be several volunteer papers, which will doubtless lead to much interesting discussion.

After adjournment the visiting delegates were entertained by the local physicians at a sumptuous oyster supper, spread by the popular caterer, Mons. Simon—"The first of the season, fine fat fellows fresh from Corpus Christi," and it is needless to say the weary M. D's did ample justice to the spread.

N, B.—No wine, yet all were happy.

